# Polo-Like Kinase 1 Directs Assembly of the HsCyk-4 RhoGAP/Ect2 RhoGEF Complex to Initiate Cleavage Furrow Formation

**DOI:** 10.1371/journal.pbio.1000110

**Published:** 2009-05-26

**Authors:** Benjamin A. Wolfe, Tohru Takaki, Mark Petronczki, Michael Glotzer

**Affiliations:** 1 Department of Molecular Genetics and Cell Biology, The University of Chicago, Chicago, Illinois, United States of America; 2 Cell Division and Aneuploidy Laboratory, Cancer Research UK London Research Institute, Clare Hall Laboratories, Hertfordshire, United Kingdom; Dana-Farber Cancer Institute, United States of America

## Abstract

To complete cell division with high fidelity, cytokinesis must be coordinated with chromosome segregation. Mammalian Polo-like kinase 1, Plk1, may function as a critical link because it is required for chromosome segregation and establishment of the cleavage plane following anaphase onset. A central spindle–localized pool of the RhoGEF Ect2 promotes activation of the small GTPase RhoA, which drives contractile ring assembly at the equatorial cortex. Here, we have investigated how Plk1 promotes the central spindle recruitment of Ect2. Plk1 phosphorylates the noncatalytic N terminus of the RhoGAP HsCyk-4 at the central spindle, creating a phospho-epitope recognized by the BRCA1 C-terminal (BRCT) repeats of Ect2. Failure to phosphorylate HsCyk-4 blocks Ect2 recruitment to the central spindle and the subsequent induction of furrowing. Microtubules, as well as the microtubule-associated protein (MAP) Prc1, facilitate Plk1 phosphorylation of HsCyk-4. Characterization of a phosphomimetic version of HsCyk-4 indicates that Plk1 promotes Ect2 recruitment through multiple targets. Collectively, our data reveal that formation of the HsCyk-4-Ect2 complex is subject to multiple layers of regulation to ensure that RhoA activation occurs between the segregated sister chromatids during anaphase.

## Abstract 2

Polo-like kinase 1 promotes assembly of the contractile ring that divides a cell in two by creating a docking site for the RhoA activator Ect2 on the Cyk-4-containing centralspindlin complex at the midzone of the mitotic spindle.

## Author Summary

The plane of cell division in animal cells is determined by the position of the mitotic spindle during early anaphase, but the molecular signaling that leads to proper formation of the division plane is not fully understood. The actin- and myosin-rich contractile ring, which physically divides a cell in two, localizes to the presumptive division plane through the local activation of a molecular switch protein, RhoA. RhoA is activated by Ect2, which binds to the protein complex centralspindlin found on microtubules in the vicinity of the division plane (the midzone microtubules). One critical component of centralspindlin is Cyk-4, a putative negative regulator of RhoA. Here, we have analyzed the mechanisms that are responsible for targeting the RhoA activator Ect2 to the midzone microtubules. We show that Polo-like kinase 1 (Plk1), in part through the microtubule-associated protein Prc1, phosphorylates Cyk-4. Ect2 binds to phosphorylated Cyk-4 and is then able to activate RhoA and induce proper formation of the contractile ring. Our study therefore has elucidated important details of the signaling cascade in animal cells that ensures proper division-plane formation.

## Introduction

Cell division requires crosstalk between various cell cycle regulatory proteins and the actomyosin and microtubule cytoskeletons. The small GTPase RhoA lies at the interface between these cytoskeletal systems, and its activation at the equatorial cortex following chromosome segregation is a critical step in the specification of the division plane [1]. RhoA activation leads to a dramatic reorganization of the actomyosin cytoskeleton underneath the plasma membrane to form a contractile network necessary for cell cleavage. The spatial regulation of RhoA activation is largely dictated by the microtubule cytoskeleton, through the combined action of microtubule asters and a set of interpolar microtubule bundles termed the central spindle [2]. The central spindle forms between the divided sister chromatids and acts as a signaling hub, integrating positional and temporal cues to facilitate activation of RhoA at the equatorial cortex [3].

The centralspindlin complex, a heterotetramer consisting of the kinesin-like protein Mklp1 (UniProt Q02241) and the RhoGAP HsCyk-4/MgcRacGAP (hereafter referred to as HsCyk-4 [UniProt Q9H0H5]), is required for assembly of the central spindle [4],[5]. In addition to its role in assembly, HsCyk-4 recruits the RhoGEF Ect2 (UniProt Q9H8V3) to the central spindle [6]–[9]. HsCyk-4 binds directly to the noncatalytic N terminus of Ect2 in a phosphorylation-dependent manner [6], via a region possessing BRCA1 C-terminal (BRCT) repeats. Because the Ect2 N terminus has been proposed to associate with and inhibit the activity of its C-terminal GEF domain [10], HsCyk-4 binding may facilitate both targeting and activation of the exchange factor for RhoA. Consistent with this model, depletion of either Ect2 or HsCyk-4 prevents RhoA-dependent cortical contractility [6]–[9]. Hence, formation of the Ect2–HsCyk-4 complex represents a critical step in cleavage plane specification, linking positional information from microtubules with cortical actomyosin contractility through RhoA activation. Because elevated Cdk1–cyclin B activity prevents HsCyk-4 and Ect2 binding [6],[7], this association is tightly controlled with respect to the cell cycle such that it occurs only during late mitosis.

The mammalian Polo-like kinase Plk1 (UniProt P53350) was shown recently through a complementary series of chemical genetic and small molecule inhibitor-based studies to be an essential activator of RhoA [11]–[14]. Inhibition of Plk1 prevents Ect2 association with HsCyk-4 and blocks its recruitment to the central spindle [11]–[14], suggesting that Plk1 might serve as a stimulatory kinase. Consistent with this, Plk1 phosphorylates Ect2 in vitro, and this phosphorylation may affect its exchange activity [15].

The functions of Polo-like kinase family members (Plks) are modulated through their subcellular distribution. Plks are recruited to various cellular sites through recognition of a phosphorylated, or primed, substrate [16]. Specifically, a C-terminal Polo-box domain (PBD) binds to a priming phosphorylation site, which serves to localize and locally activate the Plk [17],[18]. The PBD of Plk1 interacts with hundreds of mitotic proteins, each with varying affinities, to control different aspects of cell division [19]. PBD binding to the MAP Prc1 (UniProt O43663) is reported to be the primary anchor for Plk1 at the central spindle [20]. However, additional PBD binding sites independent of Prc1 are present to ensure the anaphase concentration of Plk1 at the central spindle [15],[19],[21],[22]. Here, we have examined the mechanism by which Plk1 stimulates association between Ect2 and HsCyk-4 during anaphase to trigger the onset of cytokinesis. Our data indicate that Plk1 has multiple functions in RhoA activation, part of which can be explained through its phosphorylation of HsCyk-4, and that these functions are enhanced through its targeting to the central spindle.

## Results

**Figure 1:**
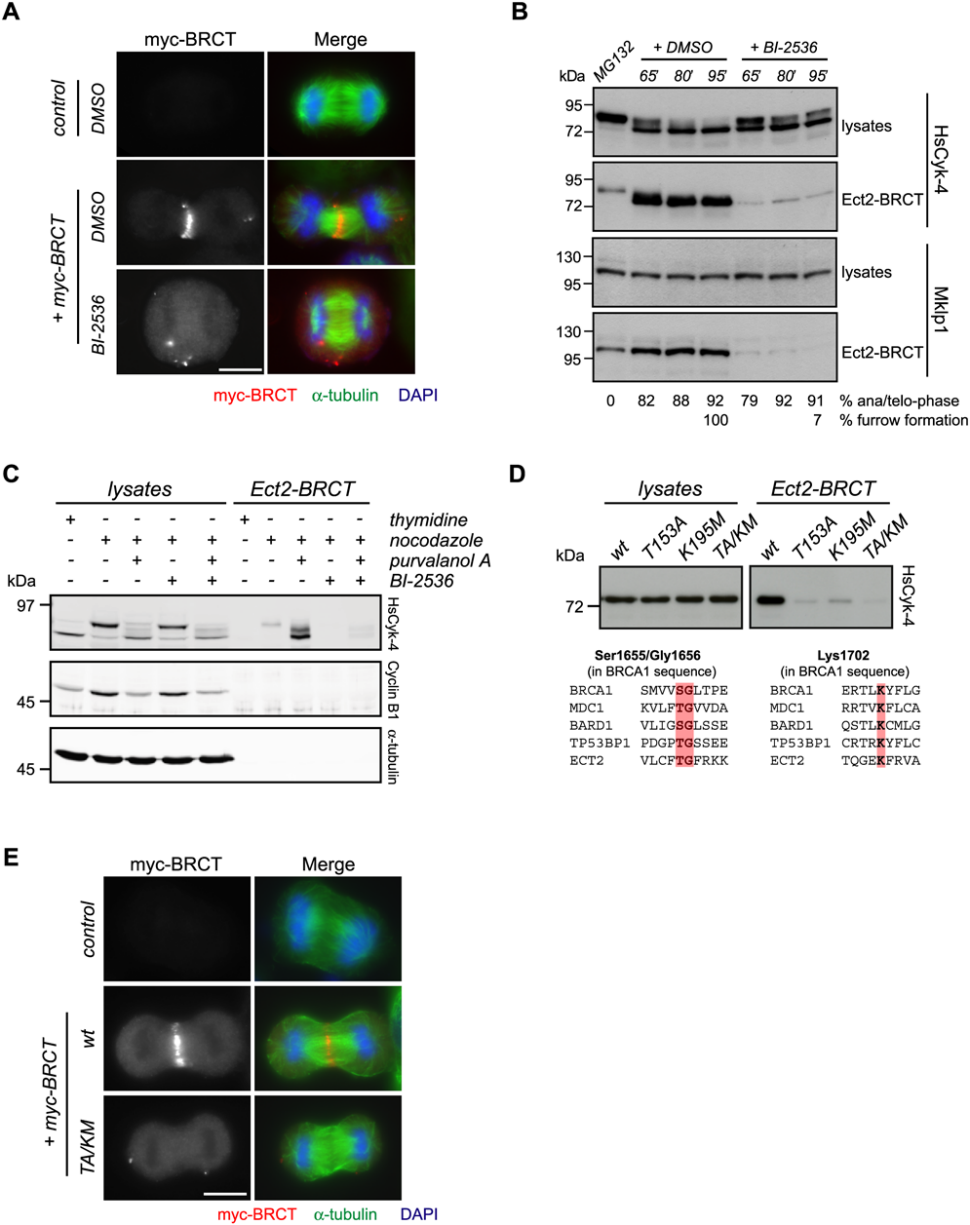
Inhibition of Plk1 prevents association of HsCyk-4 with Ect2–BRCT. (A) HeLa cells transfected with H_2_O (control) or CMV-Myc-Ect2-BRCT (myc-BRCT) were synchronized in anaphase using an MG132 arrest/release protocol. Following treatment with 100 nM BI-2536 or DMSO, cells were fixed and stained with antibodies to Myc and α-tubulin, and DNA was stained with DAPI. (B) HeLa cells, released into anaphase from an MG132 block, were treated with 100 nM BI-2536 or DMSO and harvested at the time points indicated above the lanes. Lysates and Ect2-BRCT–bound fractions were probed with antibodies to HsCyk-4 and Mklp1. The percentages of anaphase and telophase cells and of cells with ingressing furrows are indicated below the corresponding lanes. (C) HeLa cells arrested in prometaphase with nocodazole were treated with DMSO, 22.5 µM purvalanol A, 100 nM BI-2536, or 22.5 µM purvalanol A + 100 nM BI-2536 for 30 min. Lysates (∼5%) and Ect2-BRCT bound fractions were probed with antibodies to HsCyk-4, cyclin B1, and α-tubulin. (D) Lysates were prepared from HeLa cells synchronized by nocodazole block, incubated with the indicated immobilized derivatives of Ect2-BRCT, washed, and separated on SDS-PAGE. Lysates (∼5%) and Ect2-BRCT bound fractions were probed with antibodies to HsCyk-4 (upper blots). Sequence alignment showing conservation of phosphate coordinating residues in the indicated BRCT-domain containing proteins (lower sequences). (E) HeLa cells were transfected with vector (control), CMV-Myc-Ect2-BRCT (wt), or CMV-Myc-Ect2-BRCT T153A/K195M (*TA/KM*). Cells were fixed 8 h after release from thymidine, and stained with DAPI and antibodies to Myc and α-tubulin. Scale bars in this and all other figures represent 10 µm.

Chemical genetic and small-molecule–based inhibition of Plk1 kinase activity prevents both Ect2 association with HsCyk-4 and its recruitment to the central spindle [11]–[14]. Therefore, we sought to understand the mechanism whereby Plk1 regulates formation of this complex. We have shown previously that the Ect2 N terminus (Ect2-BRCT) is sufficient to localize to the central spindle [6]. Using the localization of this fragment as an assay, we tested the possibility that Plk1 may relieve the autoinhibition of Ect2, freeing the N terminus to associate with HsCyk-4. This model predicts that the central spindle localization of Ect2-BRCT would be independent of Plk1 activity. However, the Plk1 inhibitor BI-2536 [23] disrupted Ect2-BRCT localization to the central spindle in 98% of anaphase HeLa cells compared with 8% of DMSO-treated control cells (*n* = 50 for each) (Figure 1A), suggesting that relieving Ect2 autoinhibition is not the sole function of Plk1 in controlling HsCyk-4–Ect2 complex formation.

In addition to localizing to the central spindle when expressed in cells, a recombinant form of Ect2-BRCT associates with endogenous HsCyk-4 from mitotic lysates in a phosphorylation-dependent manner [6]. We therefore used this assay to ask if Plk1 activity is required for this association. First, HsCyk-4 association with Ect2-BRCT was analyzed in lysates from HeLa cells synchronously released from a metaphase block. Although only weak association was detected in metaphase, HsCyk-4, as well as its binding partner, Mklp1, were precipitated in abundance when the vast majority of control cells were in anaphase or telophase (Figure 1B). In contrast, BI-2536 potently inhibited the ability of recombinant Ect2-BRCT to precipitate either HsCyk-4 or Mklp1 (Figure 1B), suggesting that Plk1 regulates complex formation via HsCyk-4. To confirm this result, nocodazole-arrested cells were forced to exit mitosis through application of the Cdk inhibitor purvalanol A. Treatment of prometaphase cells with purvalanol A induces assembly of ectopic cleavage furrows in the absence of chromosome segregation in a manner dependent upon known cytokinesis regulators [24]. Cdk1 inhibition led to a dramatic increase in the amount of HsCyk-4 precipitated by Ect2-BRCT (Figure 1C). Concurrent application of BI-2536 and purvalanol A potently blocked HsCyk-4 precipitation by Ect2-BRCT and abrogated ectopic cleavage furrowing (Figure 1C) [12].

Dephosphorylation of mitotic lysates prevents HsCyk-4 precipitation by Ect2–BRCT [6]. Hence, we examined whether the BRCT domains of Ect2 act as a bona fide phosphobinding module. Mutation of residues within the BRCT domains of Ect2 analogous to those that coordinate phosphate in BRCA1 and MDC1 [25] abolished precipitation of HsCyk-4 from mitotic lysates (Figure 1D), confirming that Ect2's BRCT domains function as a phosphobinding module akin to those previously analyzed. Consistent with these biochemical findings, mutation of these two conserved phosphopeptide coordinating residues abolished targeting of the Ect2-BRCT domain to the central spindle in anaphase (Figure 1E).

**Figure 2:**
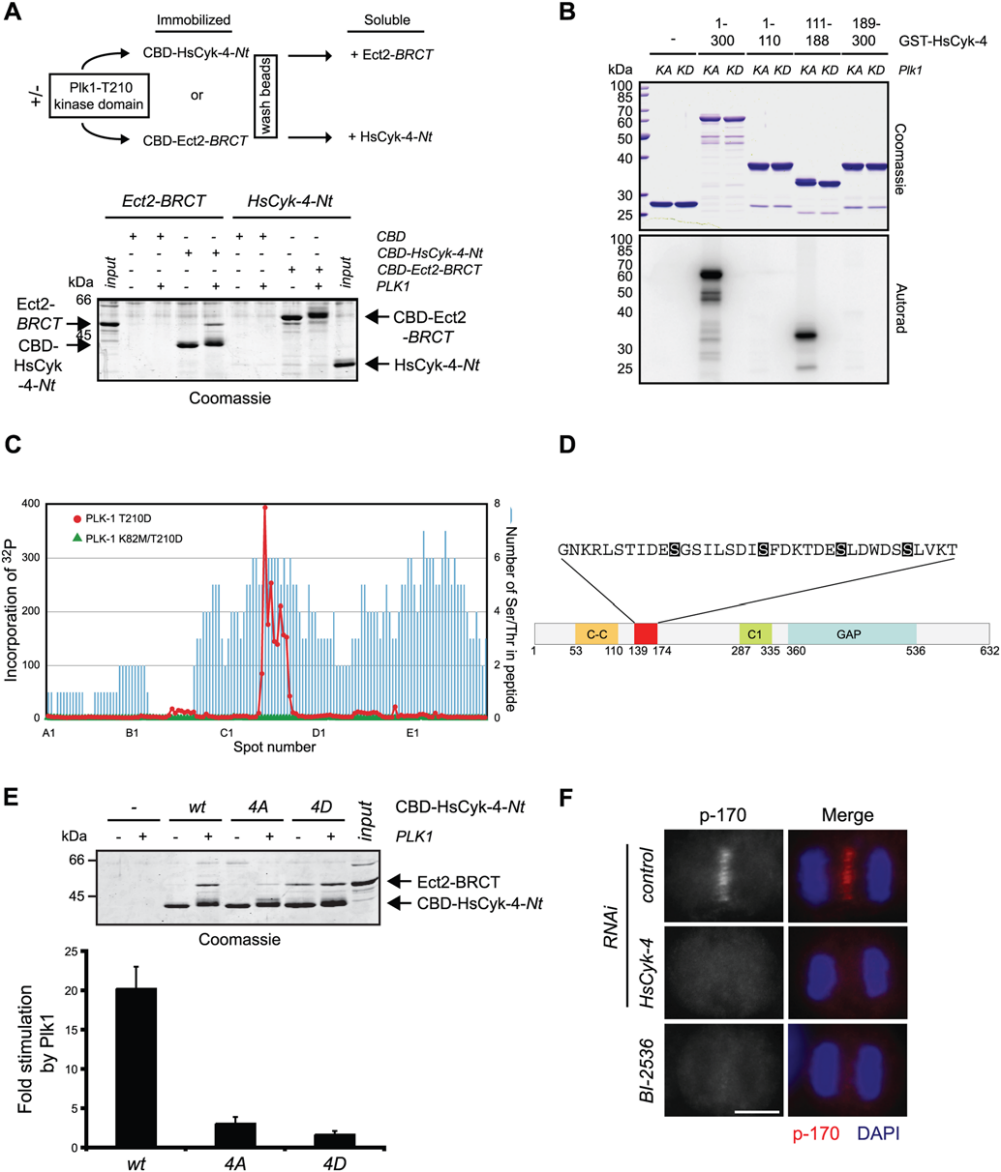
Plk1 phosphorylates multiple serine residues in HsCyk-4-Nt to stimulate association with Ect2-BRCT. (A) Schematic of experimental design (upper diagram). Recombinant HsCyk-4-Nt or Ect2-BRCT fused to CBD was incubated in the presence (+) or absence (−) of Plk1-T210D kinase domain and ATP. Soluble HsCyk-4-Nt or Ect2-BRCT was added to the washed beads, mixed, and then separated on SDS-PAGE and Coomassie stained for visualization. Input represents 50% of the total soluble protein incubated with immobilized protein. (B) Immobilized GST and a series of GST–HsCyk-4 truncated proteins as indicated were incubated with full-length recombinant Plk1 T210D (KA) or Plk1 K82M/T210D (KD) and [γ-^32^P] ATP. Proteins were separated on SDS-PAGE and Coomassie stained for visualization. Incorporation of ^32^P was visualized by autoradiography. (C) Peptide arrays containing 142 18-mer peptides covering amino acids 1–300 of HsCyk-4 were incubated with recombinant full-length Plk1 T210D or kinase-dead Plk1 K82M/T210D and [γ-^32^P] ATP. Incorporated ^32^P was visualized by autoradiography and the intensity at each peptide spot was quantified and plotted relative to the position of the peptide across the array. (D) Schematic representation of HsCyk-4 functional domains (C-C, coiled-coil domain; C1, C1 domain; GAP, GTPase activating protein domain). The red box indicates the predicted Plk1 target peptide sequence identified from the array analysis (amino acids 139–174). Within this region, Ser149, Ser157, Ser164, and Ser170 are shaded to indicate those residues mutated in the HsCyk-4 derivatives analyzed in (E). (E) Recombinant HsCyk-4-Nt and indicated derivatives fused to CBD were assayed for Plk1 phosphorylation and Ect2-BRCT binding as in (A). Fold stimulation (± standard error of the mean) of Ect2-BRCT binding to HsCyk-4-Nt upon Plk1 phosphorylation was determined from at least three independent experiments (bar graph). (F) HeLa cells transfected with H_2_O (control) or siRNA to deplete endogenous HsCyk-4 were synchronized in anaphase using an MG132 arrest/release protocol. Following treatment with 100 nM BI-2536 or DMSO, cells were fixed and stained with phospho-Ser170 antibodies, and DNA was stained with DAPI.

Our previous findings suggested that the N terminus (Nt) of HsCyk-4 could associate with Ect2-BRCT in the absence of other proteins, albeit with relatively low affinity under nonstringent conditions [6]. In vitro, Plk1 has the ability to phosphorylate both HsCyk-4-Nt and Ect2-BRCT (Figure 2A and 2B) [15]. Using purified recombinant proteins and more stringent buffer conditions, we tested whether Plk1 phosphorylation of HsCyk-4-Nt or Ect2-BRCT could stimulate association with its respective binding partner. Although Plk1 phosphorylated both proteins to high stoichiometry as evidenced by a gel mobility shift, only phosphorylation of HsCyk-4-Nt enhanced the association with Ect2-BRCT (Figures 2A and S1A).

HsCyk-4-Nt contains seven consensus motifs for Plk1 phosphorylation (E/D-X-S/T-φ) [26]. To determine which site(s) Plk1 phosphorylates, we utilized two complementary in vitro approaches. First, truncations of HsCyk-4-Nt fused to glutathione-S-transferase (GST) were used as substrates for Plk1. Of the various truncations tested, Plk1 preferentially phosphorylated the fragment encompassing amino acids 111–188 (Figure 2B). An array of HsCyk-4 N-terminal peptides was used to confirm this result and more precisely define the region of Plk1 phosphorylation. Only peptides containing amino acids 139–174 of HsCyk-4 were strongly phosphorylated by Plk1 (Figures 2C and S2A). Within this region, four putative Plk1 phosphorylation sites (Ser149, Ser157, Ser164, and Ser170) are well conserved across species and are clustered in a region downstream of the coiled-coil domain and upstream of the C1 domain (Figure 2D). Two of these residues were previously identified in a proteomic screen for phosphorylated mitotic spindle proteins (Ser164 and Ser170) [27]. Mutation of any of these four residues alone was insufficient to prevent Plk1-stimulated binding (Figure S1B). Therefore, these residues were mutated, in combination, to nonphosphorylatable Ala residues and assayed for Plk1-stimulated association with Ect2. In vitro phosphorylation of HsCyk-4-4A (amino acids 111–188) by Plk1 was dramatically reduced, to below 5% of the wild-type fragment (Figure S2B). Importantly, mutation of these four residues effectively disrupted the Plk1-mediated stimulation of HsCyk-4–Ect2-BRCT complex formation (14.9%±8.3% of wild-type association) (Figure 2E). Conversely, mutation of these four serine residues to Asp (HsCyk-4-4D) to mimic the phosphorylated state generated a form of HsCyk-4 capable of associating with Ect2-BRCT even in the absence of Plk1 phosphorylation (Figure 2E). Whereas Plk1 phosphorylation of HsCyk-4-wt stimulated association with Ect2-BRCT 20.2±2.8-fold, HsCyk-4-4A and HsCyk-4-4D produced fold increases of only 3.1±0.9 and 1.7±0.5, respectively, suggesting that the identified residues account for the vast majority of Plk1-mediated stimulation. Yeast two hybrid analysis confirmed that HsCyk-4-4D possessed an increased affinity for Ect2-BRCT (Figure S3). We conclude that Plk1 phosphorylates multiple serine residues in the N terminus of HsCyk-4 in vitro, thereby stimulating its association with Ect2.

To confirm that Plk1 phosphorylates these sites in vivo as well as to determine their spatial and temporal regulation, we obtained phosphospecific antibodies directed at Ser170 in HsCyk-4. Phospho-Ser170 antibodies specifically stained the central spindle of control, but not HsCyk-4–depleted, anaphase cells (Figure 2F). Application of BI-2536 abolished phospho-Ser170 central spindle staining, providing further evidence that Plk1 is the major kinase responsible for generating phosphorylated Ser170 in vivo.

**Figure 3:**
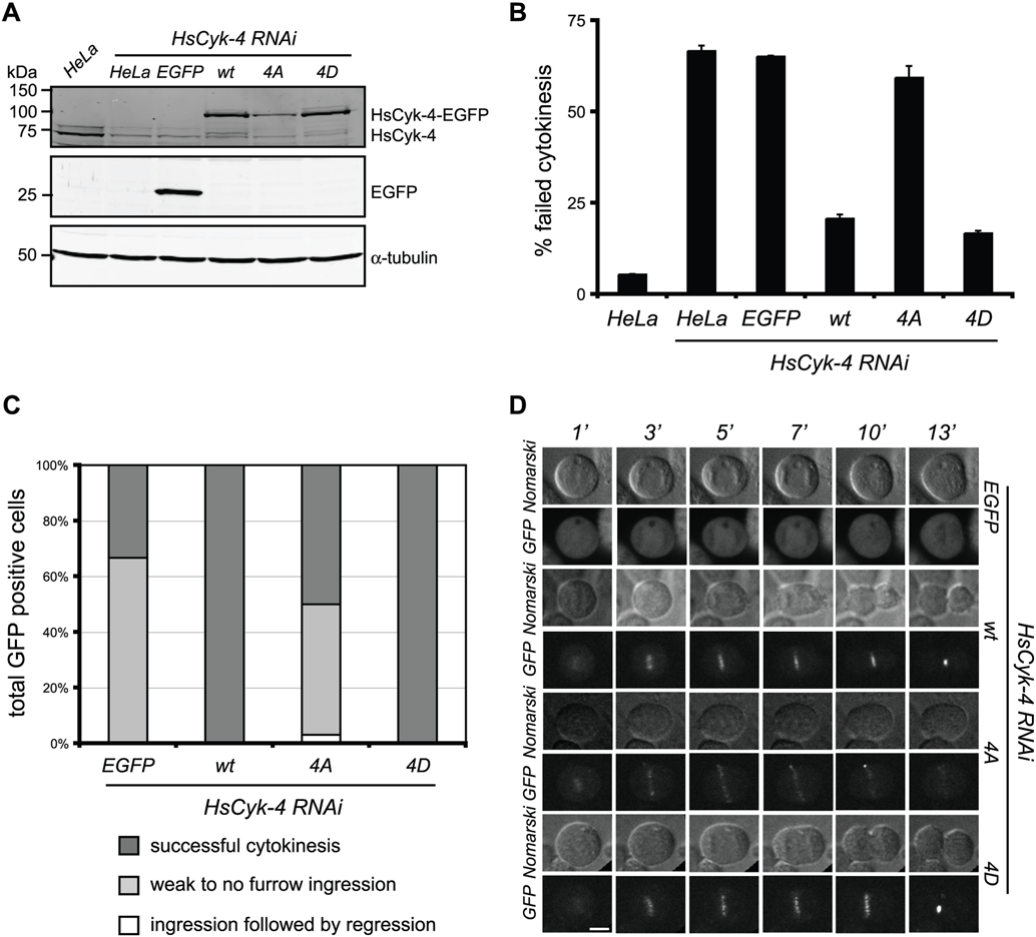
Plk1 phosphorylation of HsCyk-4 is essential for cleavage furrow formation. (A) The indicated stable HeLa cell lines and control HeLa cells were transfected with siRNA to deplete endogenous HsCyk-4. At 32 h post-transfection, lysates were prepared, separated on SDS-PAGE, and probed with antibodies to HsCyk-4, GFP, and α-tubulin. (B) The indicated stable cell lines and control HeLa cells were transfected with siRNA to deplete endogenous HsCyk-4. 32 h post-transfection cells were fixed and stained with GFP and α-tubulin antibodies, and DNA was stained with DAPI. Failed cytokinesis events were scored from the percentage of bi- or multinucleated GFP-positive interphase cells relative to the total population of GFP-positive cells (*n*≥300). Error bars represent standard deviation from two independent experiments. (C) The indicated stable cells transfected with siRNA to deplete endogenous HsCyk-4 were filmed by live video microscopy and scored for the indicated phenotypes. Only those cells expressing HsCyk-4–EGFP with a maximum intensity value above 2,000 over background fluorescence were scored for phenotype. The total number of cells scored for each cell line: EGFP, *n* = 9; wt, *n* = 15; 4A, *n* = 32; 4D, *n* = 23. (D) Nomarski and GFP time-lapse images from (C) are shown from early anaphase (1 min post metaphase exit) through late telophase (13 min post metaphase exit) for the indicated stable cells lines. Because of high levels of expression, EGFP stable cells were filmed using reduced exposure times.

**Figure 4:**
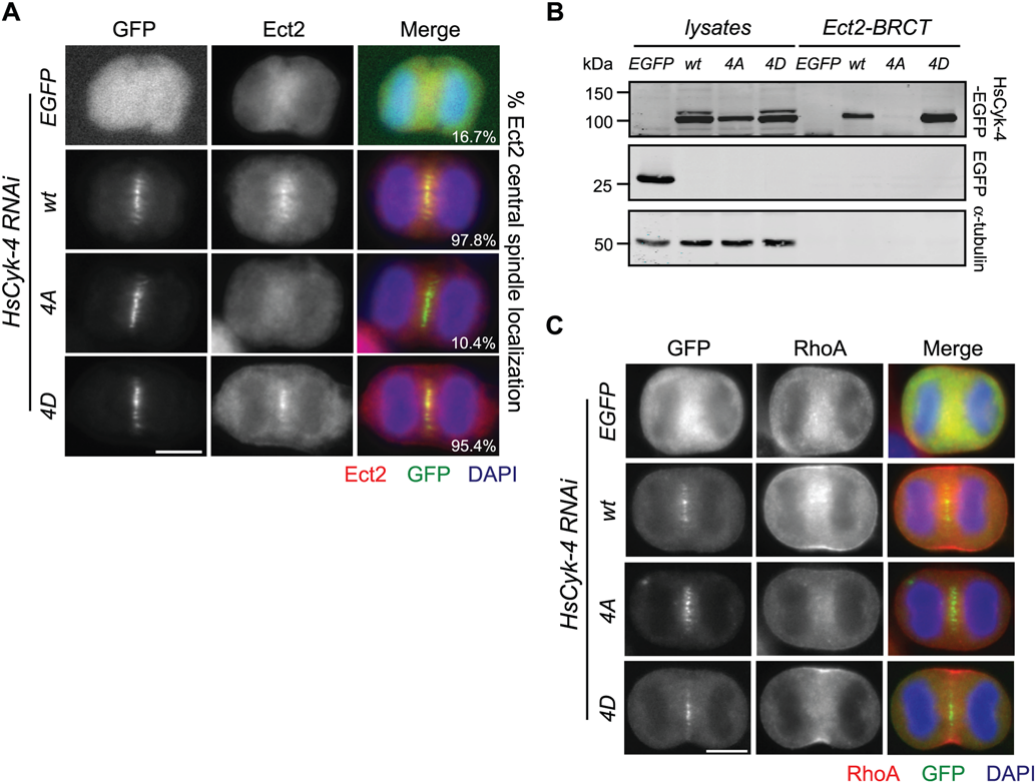
Phosphorylation of HsCyk-4 is necessary for Ect2 association. (A) The indicated stable cell lines transfected with siRNA to deplete endogenous HsCyk-4 were fixed and stained with Ect2 and GFP antibodies, and DNA was stained with DAPI. Percentages indicate the fraction of anaphase cells displaying “positive” Ect2 central spindle localization (*n*≥90 cells) as defined in Materials and Methods. (B) The indicated stable cell lines transfected with siRNA to deplete endogenous HsCyk-4 were synchronized with nocodazole, at which point 22.5 µM purvalanol A was added to the cells. Lysates and Ect2-BRCT–bound fractions were separated by SDS-PAGE and probed with antibodies to HsCyk-4, GFP, and α-tubulin. Lysate represents approximately 5% of input. (C) The indicated stable cell lines transfected with siRNA to deplete endogenous HsCyk-4 were fixed and stained with antibodies to RhoA and GFP, and DNA was stained with DAPI.

We next tested whether Plk1 phosphorylation of HsCyk-4 is essential for cleavage furrow formation. To address this, we created stable HeLa cell lines expressing RNA interference (RNAi)-resistant derivatives of HsCyk-4 fused to EGFP at its C terminus. Although variability in expression level existed within and between lines (Figure 3A), each line expressed HsCyk-4–EGFP in at least 70% of cells (Figure S4A). All HsCyk-4–EGFP derivatives, despite being expressed above endogenous levels, localized to the central spindle in cells depleted of endogenous HsCyk-4 (Figures 3D, 4A, 4C, S4A, S4B, and S4C), indicating that they retain basic functionality. Additionally, central spindle assembly remained unperturbed as indicated by proper Prc1 localization in each of the cell lines (Figure S4B). Phospho-Ser170 antibodies were used to confirm the absence of Plk1 phosphorylation in HsCyk-4-4A–EGFP stable cells (Figure S4C).

We analyzed the ability of the HsCyk-4–EGFP derivatives to rescue the cytokinesis defect associated with depletion of the endogenous protein. Cytokinesis failure was scored both in fixed cell populations (Figure 3B) and by live cell imaging of individual cells (Figure 3C and 3D). In both cases (and all subsequent experiments), cells were transfected with small interfering RNA (siRNA) to deplete endogenous HsCyk-4 for between 28 and 32 h (knockdown = 21.3%±7.4% of endogenous levels, Figure S4D). In bulk populations, a wild-type copy of HsCyk-4–EGFP was able to largely rescue the cytokinesis failure of endogenous HsCyk-4 depletion (20.7%±1.1% versus 66.7%±1.5% cytokinesis failure), whereas expression of EGFP alone failed to rescue (65.2%±0.2% cytokinesis failure) (Figure 3B). The Plk1 phospho-site mutants differed markedly in their abilities to restore efficient cytokinesis: HsCyk-4-4A–EGFP failed to rescue (59.4%±3.2% cytokinesis failure), whereas HsCyk-4-4D–EGFP rescued to a similar extent as wild type (16.7%±0.7% cytokinesis failure) (Figure 3B). In individual live cells depleted for endogenous HsCyk-4, expression of EGFP alone or HsCyk-4-4A–EGFP failed to restore cleavage furrow formation in 67% (*n* = 9) and 47% (*n* = 32) of cells, respectively (Figure 3C and 3D). In contrast, 100% of HsCyk-4-wt–EGFP (*n* = 15) and HsCyk-4-4D–EGFP (*n* = 23) expressing cells formed proper cleavage furrows and successfully completed cytokinesis. Similar results were obtained from cells cotransfected with HsCyk4 siRNA and HsCyk-4-EGFP-wt/-4A/-4D expression plasmids (Figure S5A). We conclude that phospho-site mutants of HsCyk-4 that fail to associate with Ect2 in vitro block cleavage furrow formation in vivo.

To more fully characterize the cytokinetic defect caused by the nonphosphorylatable allele of HsCyk-4, we examined the localization of critical cytokinetic regulators during anaphase in individual fixed cells. As was the case with all single-cell experiments, only cells expressing the transgenes at comparable levels were scored for phenotypic analysis (Figure S6B and S6D). First, we examined Ect2 localization in cells expressing HsCyk-4–EGFP derivatives. Although Ect2 was recruited to the central spindle by HsCyk-4-wt–EGFP and HsCyk-4-4D–EGFP during anaphase (97.8% and 95.4%, respectively, see Figure S6A for method of assessing localization), a positive Ect2 central spindle signal was not detected in nearly 90% (*n* = 96) of anaphase cells expressing HsCyk-4-4A–EGFP (Figures 4A, S6A, and S6B). Similarly, while recombinant Ect2-BRCT precipitated HsCyk-4-wt–EGFP and HsCyk-4-4D–EGFP from purvalanol A–treated cells, Ect2-BRCT failed to precipitate either EGFP alone or detectable amounts of HsCyk-4-4A–EGFP when stably expressed (Figure 4B). To exclude the possibility that the reduced level of HsCyk-4-4A–EGFP expression precluded our ability to detect its association with Ect2-BRCT, we transiently transfected HsCyk-4–EGFP derivatives to approximately equal levels and confirmed the absence of detectable association of HsCyk-4-4A–EGFP with recombinant Ect2-BRCT in purvalanol A-treated cells (Figure S5B). Consistent with its central role in cleavage furrow formation, RhoA localization to the equatorial cortex was perturbed in cells stably expressing HsCyk-4-4A–EGFP (Figure 4C). To quantitatively examine a requirement for Plk1 phosphorylation of HsCyk-4 in RhoA activation, we examined the localization of Anillin, a factor that requires active RhoA signaling for its localization to the equatorial cortex [7],[28]. Cortical accumulation of Anillin was disrupted in nearly half of all anaphase cells stably expressing HsCyk-4-4A–EGFP (Figure S6C and S6D). We conclude that Plk1 phosphorylation of the N terminus of HsCyk-4 is necessary to mediate association with Ect2 and promote RhoA activation in vivo. The phenotype associated with HsCyk-4-4A–EGFP expression is highly reminiscent of Plk1 inhibition [11]–[14], suggesting that, with respect to cleavage furrow formation, HsCyk-4 is a relevant target of Plk1's kinase activity.

**Figure 5:**
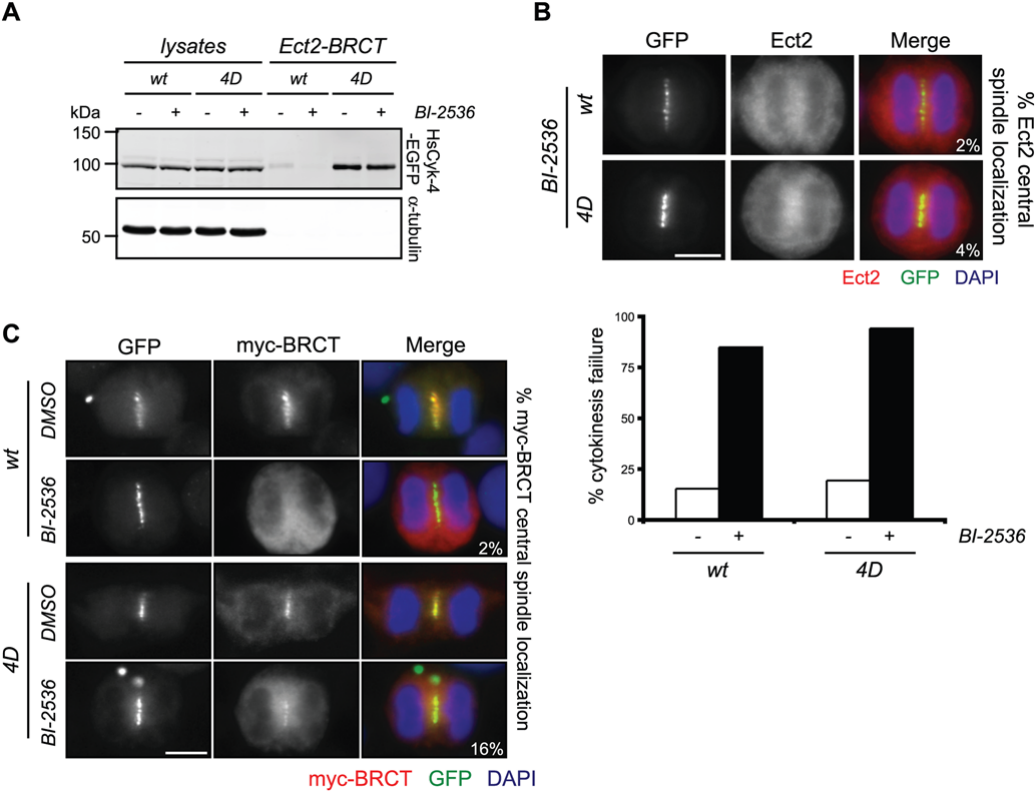
Plk1 utilizes multiple mechanisms to promote Ect2 central spindle localization. (A) The indicated stable cell lines transfected with siRNA to deplete endogenous HsCyk-4 were synchronized with nocodazole, at which point 22.5 µM purvalanol A was added to the cells in the presence or absence of 100 nM BI-2536. Lysates and Ect2-BRCT–bound fractions were separated by SDS-PAGE and probed with antibodies to HsCyk-4 and α-tubulin. Lysate represents approximately 5% of input. (B) The indicated stable cell lines were transfected with siRNA to deplete endogenous HsCyk-4 and synchronized in anaphase using an MG132 arrest/release protocol. Following treatment with 100 nM BI-2536, cells were fixed and stained with GFP and Ect2 antibodies, and DNA was stained with DAPI (upper images). Percentages indicate the fraction of anaphase cells displaying positive central spindle accumulation of Ect2 (*n*≥50 cells). The indicated stable cell lines transfected with siRNA to deplete endogenous HsCyk-4 were synchronized using an MG132 arrest/release protocol (lower bar graph). Following treatment with DMSO (−) or 100 nM BI-2536 (*+*) for 12–16 h, cells were fixed and stained with antibodies to GFP and α-tubulin, and DNA was stained with DAPI. Failed cytokinesis events were scored from the percentage of bi- or multinucleated GFP-positive interphase cells relative to the total GFP-positive population (*n*≥100). (C) The indicated stable cell lines cotransfected with CMV-Myc-Ect2-BRCT (myc-BRCT) and siRNA to deplete endogenous HsCyk-4 were synchronized in anaphase using an MG132 arrest/release protocol. Following treatment with 100 nM BI-2536 or DMSO, cells were fixed and stained with antibodies to Myc and α-tubulin, and DNA was stained with DAPI. Percentages indicate the fraction of anaphase cells displaying proper Ect2-BRCT central spindle localization (*n*≥50 cells).

If the primary function of Plk1 in generating the HsCyk-4-Ect2 complex is to phosphorylate HsCyk-4, then HsCyk-4-4D–EGFP should be able to associate with Ect2 in the absence of Plk1 kinase activity. To test this possibility, wild type and HsCyk-4-4D–EGFP stable cells were induced to exit mitosis with addition of purvalanol A in the presence and absence of BI-2536. HsCyk-4-4D–EGFP retained the ability to be precipitated by Ect2-BRCT despite the presence of BI-2536, whereas the association of wild-type HsCyk-4–EGFP with Ect2–BRCT was sensitive to the inhibitor (Figure 5A). Surprisingly, although HsCyk-4-4D–EGFP coprecipitated with Ect2-BRCT in the presence of BI-2536, endogenous Ect2 was not recruited to the central spindle during anaphase of BI-2536 treated HsCyk-4-4D–EGFP cells (Figure 5B, upper images). As a likely consequence, HsCyk-4-4D–EGFP expression did not prevent BI-2536-induced cytokinesis failure (Figure 5B, lower images). These data suggest that Plk1 likely phosphorylates multiple targets essential for cytokinesis, and that the regulated association between HsCyk-4 and Ect2 at the central spindle requires other Plk1-dependent steps in addition to HsCyk-4 phosphorylation. Because Plk1 is known to phosphorylate the C terminus of Ect2 [15], we asked whether the Ect2-BRCT fragment could localize independently of Plk1 activity in HsCyk-4-4D–EGFP cells. Whereas less than 2% of cells expressing Myc-tagged Ect2-BRCT localized the truncated protein to the central spindle in BI-2536-treated HsCyk-4-wt–EGFP expressing cells, those expressing HsCyk-4-4D–EGFP retained Ect2-BRCT localization in 16.2%±7.1% of BI-2536 treated anaphase cells (Figure 5C). We conclude that while Plk1 phosphorylation of the N terminus of HsCyk-4 is necessary and to a certain extent sufficient to mediate association with Ect2-BRCT, other Plk1-dependent events, occurring at least partially through the C terminus of Ect2, are necessary for its recruitment to the central spindle.

**Figure 6:**
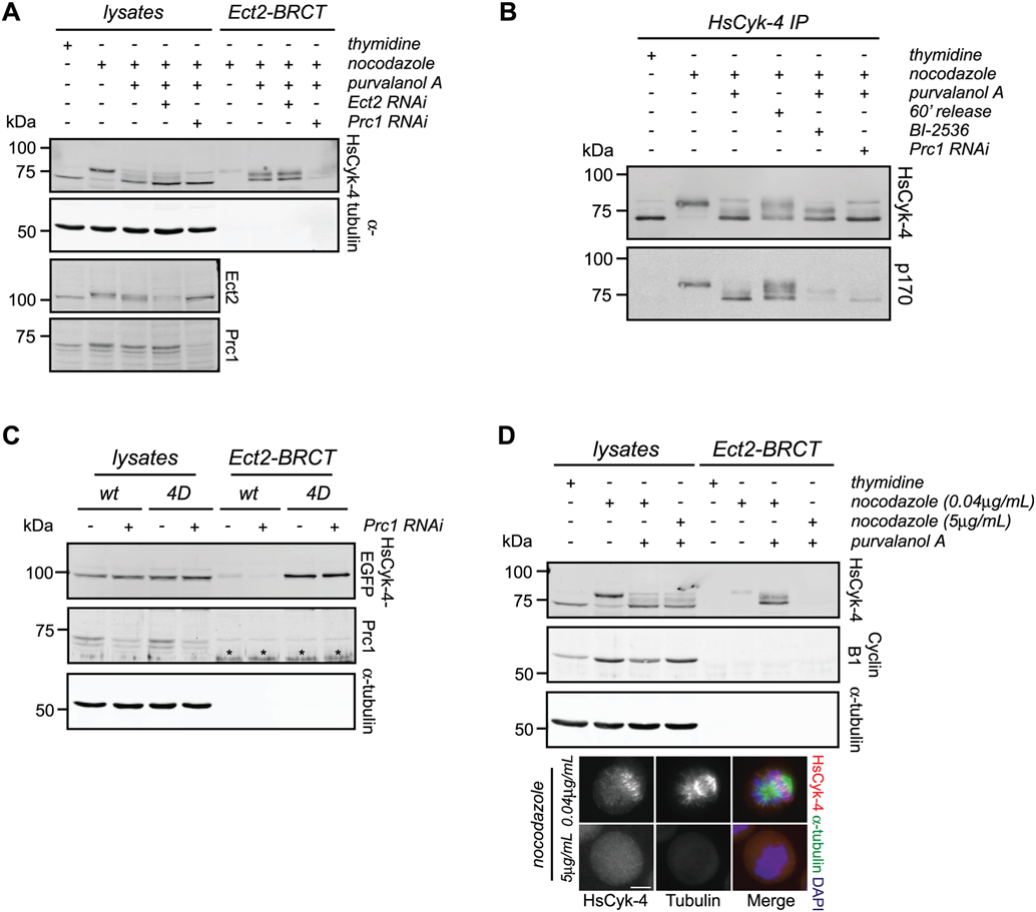
Prc1 is required for Plk1-mediated stimulation of the HsCyk-4-Ect2 complex. (A) Control HeLa cells, or those transfected with siRNA to deplete endogenous Ect2 and Prc1, were synchronized in S-phase with thymidine or in prometaphase with nocodazole. Cells were treated with DMSO or 22.5 µM purvalanol A. Lysates and Ect2-BRCT–bound fractions were separated on SDS-PAGE and probed with antibodies to HsCyk-4, α-tubulin, Ect2, and Prc1. Lysate represents approximately 5% of input. (B) HeLa cells were either mock transfected or transfected with siRNA to deplete Prc1, and synchronized in prometaphase with nocodazole. Cells were released from the prometaphase block by either addition of 22.5 µM purvalanol A or replacement with fresh medium and harvested 60 min post-release. Lysates and HsCyk-4 immunoprecipitates were separated on SDS-PAGE. Blots were probed with antibodies to HsCyk-4 and phospho-Ser170. (C) The indicated stable cell lines transfected with siRNA to deplete endogenous HsCyk-4 and Prc1, as indicated, were synchronized in prometaphase with nocodazole, at which point cells were treated with 22.5 µM purvalanol A. Lysates and Ect2-BRCT–bound fractions were separated on SDS-PAGE and probed with antibodies to HsCyk-4, Prc1, and α-tubulin. Lysate represents approximately 5% of input. Asterisks indicate the position of cross-reacting species in recombinant CBD-Ect2-BRCT recognized by Prc1 antibodies. (D) HeLa cells were synchronized with 0.04 µg/ml nocodazole, at which point either DMSO or nocodazole was added to a final concentration of 5 µg/ml in the presence of 22.5 µM purvalanol A. Lysates and Ect2-BRCT–bound fractions were separated on SDS-PAGE and probed with antibodies to HsCyk-4, cyclin B1, and α-tubulin (upper gels). Lysate represents approximately 5% of input. HeLa cells, synchronized and treated as above, were fixed and stained with antibodies to HsCyk-4 and α-tubulin, and DNA was stained with DAPI (lower images).

Having established that HsCyk-4 phosphorylation by Plk1 is critical for cleavage furrow formation, we next investigated how this phosphorylation is regulated. Plk1 substrate binding is often mediated through binding of the PBD to a phosphorylation site on the target protein. However, we did not find appreciable amounts of HsCyk-4 associated with either endogenous Plk1 or with recombinant PBD during forced mitotic exit (Figure S7A and S7B), a time when HsCyk-4 serves as a substrate for Plk1. In contrast, the MAP Prc1 associated with endogenous Plk1 as well as recombinant PBD during forced mitotic exit (Figure S6A and S6B) and, together with Mklp2, promotes Plk1 recruitment to central spindle microtubules during anaphase [20],[21]. In addition, Prc1 colocalizes with centralspindlin and direct association with HsCyk-4 has been reported [29]–[31], raising the possibility that it might serve as an intermediary in the phosphorylation of HsCyk-4. Because Ect2-BRCT precipitates HsCyk-4 in lysates from purvalanol A–treated cells, a reaction requiring Plk1 activity (Figure 1C), we were able to ask whether Prc1 was required for efficient Ect2-BRCT precipitation of HsCyk-4. The amount of HsCyk-4 precipitated in Prc1-depleted cells (knockdown = 21.5%±5.0% of endogenous Prc1 levels) was significantly decreased relative to control cells (Figure 6A). The inability of Prc1-depleted cells to generate phosphorylated HsCyk-4 provided a molecular explanation for the failure of HsCyk-4 to associate with Ect2-BRCT (Figure 6B). Consistent with the interpretation that Prc1 functions by facilitating the phosphorylation of HsCyk-4 by Plk1, expression of HsCyk-4-4D–EGFP bypassed the requirement for Prc1 to permit HsCyk-4 precipitation by Ect2-BRCT (Figure 6C).

A direct association between Prc1 and HsCyk-4 may functionally link Plk1 kinase activity with the N terminus of HsCyk-4. To test this possibility, a mutant of Prc1 (Prc1-ST601/2AA) incapable of Plk1 recruitment to the central spindle was expressed in cells depleted for endogenous Prc1 [20]. As previously reported, Prc1-ST601/2AA did not detectably interact with Plk1 (Figure S8B). Despite the disruption in Plk1–Prc1 association, Plk1 central spindle localization was only moderately attenuated (Figure S8A), and phosphorylated HsCyk-4 on Ser170 persisted at the central spindle (Figure S8C). Although this reduced level of Plk1 at the central spindle could suffice for HsCyk-4 phosphorylation, these data suggest that Plk1 need not associate directly with Prc1 in order to phosphorylate HsCyk-4.

Alternatively, as Prc1, Plk1, and centralspindlin all concentrate on a microtubule-based scaffold, Prc1-mediated bundling of microtubules may facilitate phosphorylation of HsCyk-4 by Plk1. To test this possibility, we asked whether microtubule disruption would influence the ability of Ect2-BRCT to precipitate endogenous HsCyk-4. Low-dose nocodazole treatment (0.04 µg/ml), which only weakly disrupted the microtubule cytoskeleton and retained HsCyk-4 microtubule association, permitted robust precipitation of HsCyk-4 by Ect2-BRCT upon purvalanol A addition (Figure 6D). However, 5 µg/ml nocodazole caused full microtubule destabilization, delocalized HsCyk-4, and rendered HsCyk-4 association with Ect2-BRCT undetectable (Figure 6D), underscoring the importance of a microtubule platform for this association.

**Figure 7:**
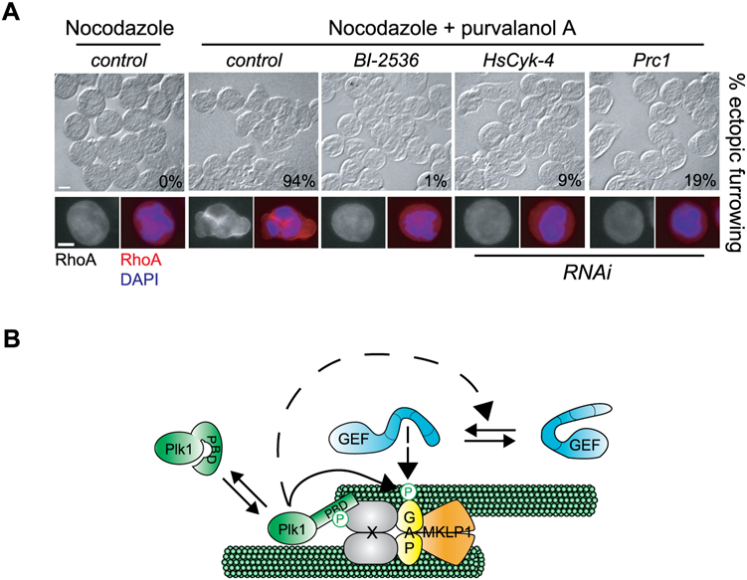
Ectopic cleavage furrowing during forced mitotic exit requires Prc1. (A) HeLa cells were either mock transfected (control) or transfected with siRNA to deplete the indicated protein and synchronized in prometaphase with nocodazole. Ectopic cleavage furrow formation and RhoA activation were induced with addition of 22.5 µM purvalanol A for 30 min. Cells were fixed and stained with antibodies to RhoA, and DNA was stained with DAPI. Percentages of cells undergoing ectopic cleavage furrow formation were determined as a function of RhoA cortical recruitment in 100 cells. (B) Proposed model. At the central spindle, microtubules are bundled through the combined efforts of the centralspindlin complex and the MAP Prc1. Factor X, which could be Prc1, Mklp2, centralspindlin, or an unknown factor, recruits Plk1 to the central spindle through association with its PBD, freeing the kinase domain to phosphorylate substrates at the central spindle (e.g., HsCyk-4, Ect2). Plk1 phosphorylation of HsCyk-4 is not sufficient for Ect2 recruitment to the central spindle, but requires other Plk1-dependent processes, at least one of which may be the relief of Ect2 autoinhibition. GAP, HsCyk-4; GEF, Ect2.

Although these data implicate both Prc1 and a microtubule scaffold as critical regulators of RhoA activation by modulating Plk1 phosphorylation of HsCyk-4, cells depleted for Prc1 can form ingressing cleavage furrows [30],[32] and those depleted of microtubules retain contractility [33], indicating that Prc1 and microtubules are not strictly essential for RhoA activation upon mitotic exit. Both prometaphase cells and cells containing monopolar spindles that are forced out of mitosis have more stringent requirements for the formation of cleavage furrows [24],[34], suggesting that the process is less robust under these circumstances. We therefore induced prometaphase cells to exit mitosis with purvalanol A and asked whether Prc1 was required for cortical RhoA localization and ectopic cleavage furrow formation. Indeed, in this context, Prc1 depletion severely compromised RhoA-induced contractility (Figure 7A). We conclude that Prc1 facilitates Plk1 phosphorylation of HsCyk-4 to allow recruitment of Ect2 to the central spindle where it can stimulate the local activation of RhoA. These data suggest that other mechanisms can compensate for the absence of Prc1 during bipolar cytokinesis.

## Discussion

The central spindle serves as a platform for the coordinated recruitment of numerous signaling proteins that regulate cytokinesis [35]. The Plk1-mediated recruitment of the RhoGEF Ect2 to the central spindle by the RhoGAP HsCyk-4 component of centralspindlin appears to be a critical step in the generation of a localized band of cortical RhoA to a region just overlying the central spindle [11]–[14]. Here, we provide a molecular mechanism whereby the HsCyk-4-Ect2 complex is formed at the central spindle following anaphase onset. By combining a reconstituted system with cell-based analyses, we have demonstrated that Plk1 promotes the phosphorylation of the N terminus of HsCyk-4, thus generating a phospho-epitope recognized by the BRCT domains of Ect2. BRCT binding may relieve Ect2 autoinhibition and facilitate activation of its intrinsic exchange activity toward RhoA. Indeed, stimulation of the HsCyk-4-Ect2 complex formation by Plk1 phosphorylation is critical for RhoA cortical localization and cleavage furrow formation. Our data also demonstrate that the role of Plk1 in stimulating HsCyk-4–Ect2 association is not limited to phosphorylation of HsCyk-4, but rather may also involve relief of Ect2 autoinhibition. Furthermore, we have identified the microtubule-associated protein (MAP) Prc1 and microtubules as critical factors facilitating Plk1 phosphorylation of HsCyk-4. Collectively, our work has elucidated a molecular basis for Plk1-regulated cleavage furrow formation and has provided further evidence that central spindle microtubules act as a crucial signaling center for cytokinesis (Figure 7B).

In order to stimulate association with the BRCT domains of Ect2, Plk1 targets multiple serine residues within the N terminus of HsCyk-4. While BRCT–phosphopeptide interactions have only been modeled with a single phosphorylated residue [36], it is possible that the tandem BRCT domains of Ect2 contact multiple phosphorylated residues. However, because mutation of the equivalent residues in Ect2 that coordinate the phosphate in MDC1 and BRCA1 [25] abrogates binding to HsCyk-4, we do not favor this possibility. Alternatively, phosphorylation of multiple residues within HsCyk-4 may lead to a conformational change that favors recognition of one particular phosphoserine by the BRCT domains of Ect2. Consistent with this, mutation of Ser157 alone within HsCyk-4 abolished association with Ect2-BRCT in mitotic lysates (unpublished data). Still, as mutation of any one of the serine residues was insufficient to prevent Plk1 stimulation of the HsCyk-4-Ect2 association in vitro, these data are not in complete accordance with one another. Instead, the most plausible explanation of these results is that each of the four phosphoserine residues within the cluster contributes to the overall binding affinity, perhaps by facilitating rebinding upon dissociation.

We have shown that the BRCT repeats of Ect2 can bind a phosphomimetic allele of HsCyk-4 (4D) from Plk1-inhibited mitotic lysates, suggesting a rather simple pathway for regulation of HsCyk-4 by Plk1. However, despite premature Ect2 association with HsCyk-4-4D in early mitotic cells (unpublished data), RhoA-induced cortical contractility was not observed. These data suggest that further steps are necessary to form a complex competent for triggering contractile events. One such step may be the removal of inhibitory Cdk1-cyclin B phosphorylations [6],[7]. Additionally, Plk1 phosphorylation of HsCyk-4 is necessary, but not sufficient, for Ect2 central spindle recruitment. As appreciable central spindle localization of isolated BRCT domains can occur in Plk1-inhibited cells expressing phosphomimetic HsCyk-4, Plk1 may also function to relieve Ect2 autoinhibition [15]. However, the recruitment of this N-terminal fragment of Ect2 is far more efficient in the presence than in the absence of Plk1 activity, suggesting the involvement of additional Plk1 substrates and additional modes of regulation. Indeed, a number of other Plk1 substrates are involved in cytokinesis, including Rock2 [19], Anillin [19], and an additional exchange factor for RhoA, MyoGEF [37]. Further work is required to define the full spectrum of Plk1 substrates required for complete activation of RhoA during cytokinesis.

Our biochemical results, obtained with synchronized cells forced out of mitosis, reveal that Prc1 and a microtubule scaffold are critical for robust generation of phosphorylated HsCyk-4. Because Prc1 recruits Plk1, and because Prc1 and HsCyk-4 colocalize at the central spindle [20],[29],[30],[32],[38],[39], it is possible that docking of Plk1 on Prc1 allows the kinase to phosphorylate HsCyk-4 while bound to Prc1. While we cannot exclude this possibility, HsCyk-4 phosphorylation persisted in cells expressing a Prc1 derivative lacking the previously defined Plk1 docking sites [20]. One important caveat is that these mutations did not completely abrogate Plk1 recruitment to the central spindle. This diminished Plk1 localization may arise through an alternative pathway, perhaps through association with Mklp1, Mklp2, or HsCyk-4 itself (Figure 7B) [19],[21],[22]. These modes of Plk1 recruitment may provide a sufficient amount of localized kinase activity to generate a biologically relevant pool of phosphorylated HsCyk-4. Because these mutations still allow microtubule bundling, Prc1 may create a scaffold of appropriately bundled microtubules on which Plk1 can target HsCyk-4 for phosphorylation. In a manner analogous to the phosphorylation of Mklp2 [21], microtubules may also enhance Plk1-mediated phosphorylation of HsCyk-4.

While in vivo analyses of bipolar cytokinesis events indicate that depletion of either Ect2, RhoA, or HsCyk4, as well as Plk1 inhibition and prevention of HsCyk-4 phosphorylation, all result in complete abrogation of cortical contractility [6]–[14],[28], depletion of Prc1 does not cause such a severe phenotype [20],[39]. In contrast, our biochemical analyses using prometaphase cells forced out of mitosis indicate that Prc1 is critical for generating significant amounts of phosphorylated HsCyk-4. Prc1 depletion in normally dividing cells results in a dramatically disorganized central spindle and a delocalization of cytokinetic factors such as Plk1 and HsCyk-4 [20],[30],[32], rendering an examination into the localization of the phosphorylated form of HsCyk-4 equivocal. Additionally, while Prc1 depletion does not block furrowing in bipolar cells, it does greatly attenuate ectopic cleavage furrowing induced by purvalanol A treatment of prometaphase cells. A similar scenario occurs in cells depleted for Mklp1. Whereas its depletion causes a furrowing defect in only 40%–50% of anaphase cells and allows generation of active RhoA [6],[8], loss of Mklp1 during forced mitotic exit almost completely abrogates the formation of ectopic cleavage furrows and the recruitment of RhoA to the cortex (unpublished data). Thus, the phenotypes resulting from a reduction in RhoA activation are likely to be context dependent, with the ectopic cleavage furrowing induced by purvalanol A perhaps being particularly dependent on events that occur on bundled microtubules [34],[40],[41].

In summary, our data reveal that many levels of protein phosphorylation regulate the activation of RhoA upon anaphase onset. The recruitment of critical central spindle components such as Prc1 and centralspindlin, as well as the formation of the HsCyk-4-Ect2 complex, are negatively regulated by Cdk1–cyclin B–mediated phosphorylation [6],[20],[39],[42],[43]. Following a decline in Cdk1–cyclin B activity at anaphase onset, microtubules are bundled and Plk1 is recruited to the central spindle through association of its PBD with Prc1 [20], Mklp2 [21], and possibly other factors [19],[22]. At the central spindle, Plk1 performs several functions critical for cleavage furrow formation, including HsCyk-4 phosphorylation and subsequent Ect2 recruitment, as well as activation of RhoA. These multiple layers of regulation conspire to ensure that activation of the cytokinetic machinery occurs at the appropriate time and place.

## Materials and Methods

### Cell Culture and Drug Treatment

Kyoto HeLa S3 cells were grown in Dulbecco's Modified Eagle Medium (DMEM) supplemented with 10% FBS, 2 mM L-glutamine, 100 U penicillin, and 0.1 mg/ml streptomycin. Stable cell lines expressing siRNA-resistant HsCyk-4–EGFP and derivatives were constructed in HeLa cells grown in DMEM containing 200 µg/ml hygromycin B. Previously described dsRNAs were used in this study to deplete endogenous HsCyk-4 [6], Ect2 [6], and Prc1 [20]. Oligofectamine (Invitrogen) and Lipofectamine-2000 (Invitrogen) were used for transfection of siRNA and plasmid DNA, respectively. Purvalanol A (Axxora) was used at 22.5 µM. Nocodazole (Sigma) was used at 0.04 µg/ml to mildly perturb the microtubule cytoskeleton and arrest cells in prometaphase, and at 5 µg/ml to fully deplete the microtubule cytoskeleton. BI-2536 (generously provided by Norbert Kraut, Boehringer Ingelheim) was used at 100 nM [23]. MG132 (Sigma) was used at 10 µM.

### Live Imaging and Immunofluorescence

To perform live cell imaging analysis, cells were grown in the Delta T4 open dish system (BiOptechs) and controlled at 37°C during the filming process. Cells were visualized using a 40×/.75 NA objective on a Zeiss Axiovert 200M equipped with a Yokogawa CSU-10 spinning-disk unit (McBain), illuminated with a 50 mW 473 nm DPSS laser (Cobolt). Single-plane multipoint acquisitions were captured every 20 s on a Cascade 512B EM-CCD camera (Photometrics) using MetaMorph (Molecular Devices) software. All images were acquired under identical conditions and scaled and processed identically (with the exception of EGFP stable cells, which were acquired using a reduced exposure). Cells were considered to be “positive” for transgene expression if the maximum intensity of cellular fluorescence was >2,000 above the background intensity. For immunofluorescence analysis, cells were grown on coverslips and fixed with methanol at −20°C for either 15 min or overnight (for Anillin, Ect2, GFP, Myc, phospho-Ser170, Plk1, and Prc1 staining) or with 10% trichloroacetic acid on ice for 15 min (for RhoA, GFP, HsCyk-4, and α-tubulin staining). Fluorescently conjugated secondary anti-mouse or anti-rabbit (Alexa 488 or 568, Molecular Probes) antibodies were used at 1∶500 dilutions. Images were collected with a Zeiss AxioImager M1 microscope using a 40×/0.75 objective. All images were acquired under identical conditions using Metamorph (Molecular Devices) software. The 16-bit images were then opened in Image J and scaled to 8-bit with a single scale for all images in a given experiment. For analysis of Ect2 localization to the central spindle, quantification was performed as follows: using raw image data acquired under identical conditions, the fluorescence intensity of Ect2 in a region of interest (ROI) containing the central spindle was quantified. From that value, we subtracted the average of ROIs immediately adjacent to the central spindle. Should the resulting value be ≥1,000, then Ect2 central spindle localization was deemed “positive.” A similar scheme was used to determine “positive” Anillin localization to the equatorial cortex. Specifically, using raw image data acquired under identical conditions, the fluorescence intensity of Anillin in a ROI containing the equatorial cortex as well as a ROI in the cytoplasm that was immediately adjacent to the equatorial cortex were quantified. When the ratio of the equatorial ROI to cytoplasmic ROI was greater than 1, then Anillin localization to the equatorial cortex was deemed “positive.”

### Cell Synchronization, Pull-Downs, and Immunoblotting

HeLa cells and stable cell lines were synchronized as described [6],[12]. Cells were either released from a nocodazole (prometaphase) block or forced to exit mitosis by addition of purvalanol A (Axxora) for 30 min. For all synchronization experiments involving RNAi, siRNA transfections took place approximately 1 h following release from the first overnight thymidine block so that the duration of RNAi was between 28 and 32 h. Lysates were prepared as described [6]. For the Ect2-BRCT and PBD precipitation experiments, approximately 650 µg of total protein was incubated with 10 µg of immobilized chitin-binding domain (CBD)–Ect2-BRCT (1–421) and 10 µg GST–PBD^+^ (construct generously provided by D. Lim and M. Yaffe, Massachusetts Institute of Technology [MIT]), respectively, for 12–15 h at 4°C with mixing. For immunoprecipitation experiments, approximately 1.5–2 mg of total protein was incubated with either 2 µg of anti-Plk1 or anti-Ect2 antibodies for 12–15 h at 4°C with mixing. Protein A-sepharose was added and mixing at 4°C was continued for an additional 45 min. All recombinant beads and immunocomplexes were washed extensively in lysis buffer and boiled in sample buffer prior to separation on SDS-PAGE. The following antibodies were used for Western blot analysis: mouse anti-HsCyk-4 (Abnova RacGAP1 1∶1,000), mouse anti-α-tubulin (Sigma DM1α 1∶10,000), mouse anti-Plk1 (Santa Cruz 1∶500), mouse anti-c-Myc (Boehringer Mannheim 9E10 1∶5,000), rabbit anti-GFP (Santa Cruz 1∶500), rabbit anti-Ect2 ([6] 1∶1,000), rabbit anti-Mklp1 ([4] 1∶1,000), rabbit anti-Prc1 (Santa Cruz 1∶500), rabbit anti-cyclin B1 (Santa Cruz 1∶500), rabbit anti-phospho-Ser170 (generously provided by P. Jallepalli, MS-KCC, 1∶1,000) goat-anti-mouse IR-680 (Invitrogen 1∶5,000), and goat-anti-rabbit IR-800 (Jackson Labs 1∶5,000). Membranes were imaged using an Odyssey scanner (Li-Cor) and bands quantified using Odyssey v2.1 software (Li-Cor).

### Peptide Array Analysis

One hundred forty-two 18-mer peptides corresponding to HsCyk-4 amino acids 1–300 and a positive control Plk1 substrate peptide (SFNDTLDFD) [44] were synthesized and spotted on cellulose membranes. The sequence of each 18-mer peptide is shifted by two residues resulting in a 16 amino acid sequence overlap. Membranes were equilibrated in kinase buffer (50 mM Tris-HCl [pH 7.5], 10 mM MgCl_2_, 1 mM EGTA, and 2 mM DTT) and incubated with 100 nM full-length recombinant Plk1 T210D or Plk1 K82M/T210D in kinase buffer with 1 µM ATP and 25 µCi [γ-^32^P] ATP at 30°C for 60 min. Reaction was terminated with 100 mM phosphoric acid, and membranes were washed extensively in 1 M NaCl and 0.5% phosphoric acid. Membranes were soaked into methanol before drying, then exposed to phosphor screen (GE Healthcare). The incorporation of ^32^P was analyzed by phosphor imager (Typhoon, GE Healthcare), and spot signal intensities were quantified using ImageQuant (GE Healthcare).

### In Vitro Binding

50 nM of purified recombinant Plk1-T210D 1–306 (generously provided by D. Lim and M. Yaffe, MIT) was used to phosphorylate 100 nM bead-bound CBD tagged HsCyk-4-Nt or Ect2-BRCT in kinase buffer (50 mM Tris-HCl [pH 7.5], 10 mM MgCl_2_, 1 mM EGTA, and 2 mM DTT) for 60 min at 30°C with rigorous mixing. Beads were washed extensively in binding buffer (20 mM HEPES [pH 7.2], 150 mM NaCl, 5 mM MgCl_2_, 1 mM DTT, 0.1% Triton X-100), and the appropriate binding partner was added in soluble form at equimolar amounts. Binding reactions were incubated for 90 min at 4°C with mixing. Beads were washed extensively in binding buffer and boiled in sample buffer prior to separation on SDS-PAGE. Bands were visualized either by Coomassie staining or by Western blot analysis.

### Recombinant Protein Expression and Purification

Ect2-BRCT (1–421) and HsCyk-4-Nt (1–288) and associated derivatives were fused to the CBD and expressed in BL21 (DE3) RIL cells. Expression was induced by the addition of 0.4 mM IPTG at 25°C for 4–5 h. Bacteria were resuspended in 10 mM HEPES (pH 7.7), 250 mM NaCl, 1 mM EGTA, 1 mM MgCl_2_, 0.1% Triton-X 100, 1 mM DTT, 0.1 mM ATP, 10 µg/ml leupeptin/pepstatin, and 1 mM phenylmethylsufonylflouride containing 0.5 mg/ml lysozyme prior to sonication. Lysates were cleared at 18,000 rpm at 4°C for 20 min in a JA.20 Beckman rotor. Prewashed chitin beads (New England Biolabs) were added to the cleared lysates and incubated for 2 h at 4°C with mixing. Following washes, CBD fusions were either maintained on beads, aliquoted, and stored at −80°C, or the fusion tag was removed by addition of recombinant Tobacco Etch Virus (TEV) overnight at 4°C with mixing, and the resulting eluate was stored in aliquots at −80°C.

Mutations Thr210 to Asp and Lys82 to Met were introduced in the cDNA of human Plk1 using site-directed mutagenesis (Quickchange II, Stratagene) to generate full-length activated (kinase active [KA]) Plk1 (T210D) and enzymatically inactive (kinase dead [KD]) Plk1 (K82M/T210D). The resulting Plk1 variants were cloned into pFastBac1 (Invitrogen) containing GST and a PreScission protease recognition sequence upstream of the cloning site. GST-Plk1 T210D and GST-Plk1 K82M/T210D were expressed in Sf-9 cells using the baculovirus system. Infected cells were incubated for 72 h, treated with 0.1 µM okadaic acid for 2 h, and then harvested. Cells were resuspended in buffer A (50 mM HEPES [pH 7.5], 150 mM NaCl, 1 mM EDTA, 2.5 mM EGTA, 0.1% NP-40, 10% glycerol, 1 mM DTT, 1 µM microcystin LR, and protease inhibitor cocktail [Roche]). After incubation in buffer A for 30 min at 4°C, cells were lysed by sonication. Lysates were centrifuged at 28,000*g* for 30 min at 4°C. GST fusion proteins purified using glutathione sepharose 4B (GE Healthcare). GST-Plk1 proteins were eluted in buffer containing 20 mM glutathione and subsequently incubated with PreScission protease (GE Healthcare) for 3 h at 4°C. Following cleavage, the reaction mixture was dialyzed three times against buffer C (50 mM Tris-HCl [pH 7.5], 150 mM NaCl, 1 mM EDTA, 10% glycerol, and 1 mM DTT). To remove GST and PreScission protease, the dialyzed fraction was incubated with glutathione sepharose 4B. The flow-through containing Plk1 was frozen in liquid nitrogen and stored at −80°C. Amount, purity, and activity of Plk1 T210D and Plk1 K82M/T210D were determined by SDS-PAGE, Coomassie brilliant blue staining, and in vitro kinase assays using casein as a model substrate (unpublished data).

### Plk1 Kinase Reactions

For labeled kinase reactions, GST and a series of HsCyk-4 fragments fused to the C terminus of GST were expressed in *E. coli* and purified using glutathione sepharose 4B (GE Healthcare). Immobilized GST or GST-HsCyk-4 protein was incubated with full-length recombinant Plk1 T210D or Plk1 K82M/T210D in kinase buffer with 50 µM ATP and 1 µCi [γ-^32^P] ATP at 30°C for 30 min. Beads were washed in PBS containing 1% Triton X-100 and boiled in SDS sample buffer. Samples were resolved by SDS-PAGE, visualized by Coomassie brilliant blue staining, and finally analyzed using a phosphor imager (typhoon, GE Healthcare).

### Yeast Two Hybrid Analysis

*S. cerevisiae* strain PJ69-4A (generously provided by K. Gould, Vanderbilt) was cotransformed by lithium acetate method with bait (pGBT9, Trp^+^) and prey (pGAD424, Leu^+^) plasmids according to standard techniques [45]. Leu^+^/Trp^+^ transformants were scored for positive interactions by serial dilution on synthetic dextrose medium lacking adenine and histidine.

## Supporting Information

Figure S1 **Plk1 phosphorylates the N terminus of HsCyk-4 to simulate association with Ect2-BRCT.** (A) Schematic of experimental design (upper diagram; same as Figure 2A). Recombinant HsCyk-4-Nt or Ect2-BRCT fused to CBD was incubated in the presence (+) or absence (−) of Plk1-T210 kinase domain and ATP. Soluble HsCyk-4-Nt or Ect2-BRCT was added to the washed beads, mixed, and then separated on SDS-PAGE and probed with antibodies to Ect2 and HsCyk-4 (lower gels). Input represents 50% of the total soluble protein incubated with immobilized protein. (B) Recombinant HsCyk-4-Nt and indicated derivatives fused to CBD were assayed for Plk1 phosphorylation and Ect2-BRCT binding as in (A). Proteins were resolved on SDS-PAGE and Coomassie stained for visualization. Input represents 50% of the total soluble protein incubated with immobilized protein.
(2.87 MB EPS)

Figure S2 **Plk1 phosphorylates N-terminal peptides of HsCyk-4 on four serine residues.** (A) Synthesized 18-mer peptides, corresponding to HsCyk-4 1–300, and control peptide were spotted on cellulose membranes. Membranes were incubated with full-length recombinant Plk1 T210D (upper panel) or Plk1 K82M/T210D (middle panel) and [γ-^32^P] ATP. Incorporation of ^32^P was visualized by autoradiography. (B) Immobilized GST, GST-HsCyk-4 (111–188), and GST-HsCyk-4 4A (111–188) were incubated with recombinant Plk1 T210D (KA) or Plk1 K82M/T210D (KD) and [γ-^32^P] ATP. Proteins were separated by SDS-PAGE and visualized by Coomassie brilliant blue staining. Incorporation of ^32^P was analyzed by autoradiography. Relative incorporation of ^32^P to GST-HsCyk-4 was quantified and is shown below the corresponding lanes.
(6.79 MB EPS)

Figure S3 **Association of HsCyk-4-Nt and Ect2-BRCT in yeast.** Strain KGY 1296 was cotransformed with the indicated bait (pGBT9) and prey (pGAD424) plasmids and selected on media lacking Trp and Leu. Cotransformed cells were serially diluted (1∶10) onto either +His +Ade medium (growth does not require reporter activation) or −His −Ade (growth requires reporter activation). EV, empty vector control.
(3.43 MB EPS)

Figure S4 **Characterization of cell lines stably expressing HsCyk-4–EGFP derivatives.** (A) The indicated stable cell lines transfected with siRNA to deplete endogenous HsCyk-4 were synchronized in anaphase using a MG132 arrest/release protocol. Cells were fixed and stained with GFP antibodies and 4′,6-diamidino-2-phenylindole (DAPI), and scored for percentage of anaphase cells possessing HsCyk-4–EGFP at the central spindle (*n* = 100). (B) The indicated stable cell lines were transfected with siRNA to deplete endogenous HsCyk-4 and synchronized in anaphase using a MG132 arrest/release protocol. Cells were fixed and stained with antibodies to GFP and Prc1, and DNA was stained with DAPI. (C) The indicated stable cell lines were either mock transfected (control) or transfected with siRNA to deplete endogenous HsCyk-4 and synchronized in anaphase using a MG132 arrest/release protocol. Cells were fixed and stained with antibodies to GFP and phospho-Ser170, and DNA was stained with DAPI. (D) Relative amounts of endogenous HsCyk-4 levels were determined in mock-transfected cells (control) and in cells transfected with siRNA to deplete endogenous HsCyk-4 for between 28 and 32 h. Lysates were prepared, separated on SDS-PAGE, and probed with antibodies to HsCyk-4 and α-tubulin as a loading control. Quantification of band intensities was performed using Odyssey v2.1 software. Standard deviation was generated from at least four independent experiments.
(7.04 MB EPS)

Figure S5 **Transiently transfected HsCyk-4-4A–EGFP fails to promote cleavage furrow formation and Ect2-BRCT association.** (A) HeLa cells cotransfected with the indicated HsCyk-4–EGFP fusions and siRNAs to deplete endogenous HsCyk-4 were filmed by live video microscopy and scored for cytokinesis failure as in Figure 3C. The total number of transfected cells scored for each cell line: EGFP, *n* = 13; wt, *n* = 12; 4A, *n* = 17; 4D, *n* = 12. (B) HeLa cells cotransfected with the indicated HsCyk-4-EGFP fusions and siRNAs to deplete endogenous HsCyk-4 were synchronized in prometaphase with nocodazole and released by addition of 22.5 µM purvalanol A. Lysates were prepared, precipitated with recombinant Ect2-BRCT, washed and separated on SDS-PAGE, and probed with antibodies to HsCyk-4 and α-tubulin. Lysates represent approximately 5% of input.
(1.69 MB EPS)

Figure S6 **Determination of Ect2 and Anillin failure to localize to the central spindle and equatorial cortex, respectively, in HsCyk-4-4A–EGFP–expressing cells.** (A) The indicated stable HeLa cell lines expressing HsCyk-4–EGFP were transfected with siRNAs to deplete endogenous HsCyk-4 and synchronized in anaphase using a MG132 arrest/release protocol. Cells were fixed and stained with Ect2 and GFP antibodies, and DNA was visualized using DAPI. Positive central spindle accumulation of Ect2 was determined as indicated in Materials and Methods. (B) A subset of the cells analyzed in Figure 4A were selected for quantification, and the intensity of Ect2 at the central spindle (quantified as above) was plotted relative to HsCyk-4–EGFP levels for the indicated stable cell lines depleted for endogenous HsCyk-4. The area above the dashed line represents those cells possessing “positive” Ect2 central spindle localization; below the dashed line represents “failed” central spindle localization. (C) The indicated stable cell lines transfected with siRNA to deplete endogenous HsCyk-4 were synchronized in anaphase using an MG132 arrest/release protocol. Cells were fixed and stained with antibodies to GFP and Anillin, and DNA was stained with DAPI. Percentage of cells with positive Anillin localization (see Materials and Methods section for assessment of “positive”) to the equatorial cortex was determined in at least 100 cells. (D) A subset of the cells analyzed in Figure S6C were chosen for quantification, and the ratio of fluorescence intensity values of Anillin at the equatorial cortex: Anillin in the cytoplasm (quantified as above) was plotted relative to HsCyk-4–EGFP levels for the indicated stable cell lines depleted for endogenous HsCyk-4. The area above the dashed line represents those cells possessing “positive” Anillin localization to the equatorial cortex; below the dashed line represents “failed” cortical localization.
(6.26 MB EPS)

Figure S7 **Plk1 associates with Prc1, but not HsCyk-4, during forced mitotic exit.** (A) HeLa cells synchronized with nocodazole were treated with DMSO or 22.5 µM purvalanol A in the presence or absence of 100 nM BI-2536. Lysates and Plk1 immunoprecipitates were separated on SDS-PAGE and probed with Plk1, Prc1, Ect2, and HsCyk-4 antibodies. (B) HeLa cells synchronized with nocodazole were treated with either DMSO, 22.5 µM purvalanol A, or 100 nM BI-2536. Lysates and PBD-bound (PBD^+^) fractions were separated on SDS-PAGE and probed with Ect2, Prc1, and HsCyk-4 antibodies (upper panels). Lysate represents approximately 5% of input. The ratio of PBD bound HsCyk-4, Ect2, and Prc1 in purvalanol A or BI-2536 treated cells over nocodazole control were plotted (lower panel).
(4.58 MB EPS)

Figure S8 **HsCyk-4 remains phosphorylated on Ser-170 in cells expressing Prc1-ST601/2AA.** (A) HeLa cells cotransfected with siRNA to deplete endogenous Prc1 and CMV-Myc-Prc1-wt (*wt*) or CMV-Myc-Prc1-ST601/2AA (*AA*) were synchronized in anaphase using an MG132 arrest/release protocol. Cells were fixed and stained with antibodies to Myc and Plk1, and DNA was stained with DAPI. (B) HeLa cells cotransfected with siRNA to deplete endogenous Prc1 and the indicated CMV-Myc-Prc1 constructs were arrested in prometaphase with nocodazole. Cells were released with addition of 22.5 µM purvalanol A for 30 min. Lysates were prepared, immunoprecipitated using Plk1 antibodies, washed, and separated on SDS-PAGE. Blots were probed with antibodies to Myc and Plk1. Asterisks indicate the position of cross-reacting species recognized by Myc antibodies. Lysate represents approximately 5% of the input. (C) HeLa cells transfected and synchronized as in (A) were fixed and stained with antibodies to Myc and phospho-170, and DNA was stained with DAPI.
(8.42 MB EPS)

## Abbreviations

BRCT: BRCA1 C-terminal
CBD: chitin-binding domain
Cdk: cyclin-dependent kinase
CMV: cytomegalovirus
DAPI: 4′,6-diamidino-2-phenylindole
DMSO: dimethyl sulfoxide
EGFP: enhanced green fluorescent protein
GFP: green fluorescent protein
GST: glutathione-S-transferase
KA: kinase active
KD: kinase dead
MAP: microtubule-associated protein
Nt: amino terminus
PBD: Polo-box domain
RNAi: ribonucleic acid interference
ROI: region of interest
SDS: sodium dodecyl sulfate
siRNA: small interfering RNA
wt: wild type
